# Editorial: Time discounting as a tool to assess addictive behaviors and other disorders

**DOI:** 10.3389/fpubh.2023.1165175

**Published:** 2023-03-17

**Authors:** Salvador Cruz Rambaud, María José Muñoz Torrecillas, Fabrizio Maturo

**Affiliations:** ^1^Department of Economics and Business, University of Almería, Almería, Spain; ^2^Faculty of Economics, Universitas Mercatorum, Rome, Italy

**Keywords:** addictive behaviors, substance abuser, drug addict, intertemporal choices, psychological analyses

Decisions in intertemporal choice have become one of the main indicators that can detect some specific addictive behaviors and other diseases. For example, a gambling addict or a substance abuser (specifically, a drug addict) may exhibit high levels of impatience in intertemporal decisions where monetary and non-monetary rewards are involved.

It is well known that intertemporal choices can be ruled by a discount function, which is the mathematical instrument able to quantify the main parameters related to decisions where delayed and interval times are present. Thus, a high level of impatience means that the rate derived from the discount function is excessive. However, an excessive discount rate may not only be the alert signal of an individual disorder. In effect, the process of intertemporal choice can exhibit other anomalies with respect to the Discounted Utility model proposed by Samuelson in 1936, such as the so-called delay, interval, magnitude, sign and improving sequence effects, or the well-known delay-speedup asymmetry.

Therefore, the main objective of the Research Topic “*Time discounting as a tool to assess addictive behaviors and other disorders*” of the journal “Frontiers in Public Health” is the analysis of certain behaviors associated with addiction and other diseases, and their effects on the parameters of the discount function fitting the indifference between intertemporal decisions. The methodology used in the Research Topic ranges from the empirical research on medical or psychological treatment of addictions and other disorders to the mathematical analysis of the discount models associated with the intertemporal processes involving reward selection. Therefore, theoretical and empirical research was welcome. To summarize, our aim is to build a bridge connecting, on the one hand, medical and psychological analyses, and, on the other hand, quantitative models derived from observed individual or group behaviors.

The Research Topic included in this special issue of “Frontiers in Public Health” could be of interest to scholars belonging to a wide variety of expertise fields, such as medicine, psychology, economics, financial mathematics, and, in general, all researchers who investigate behavioral disorders that can be related to a specific pattern in intertemporal decisions with monetary and non-monetary rewards. Societies are currently facing problems such as stress, anxiety, gambling, drug use, etc. of a wide target population. Thus, all instruments able to detect or diagnose early diseases are very interesting for public health.

The results expected from this Research Topic are very promising because there are many research teams all over the world that are working on this topic from different points of view: descriptive, quantitative, inductive, deductive, etc. In this way, this Research Topic collects all papers written around the aforementioned central topic, avoiding the inconveniences of finding an appropriate outlet to share the wide variety of expected contributions. Finally, we hope that this initiative can serve as a means for connecting fruitful ideas aimed at reaching a healthier public environment.

The paper “*Social media sites users' choice between utilitarian and informational reinforcers assessed using temporal discounting*”, by Robayo-Pinzon et al., provides a first approach to the use of the so-called Multiple Choice Procedure in social media network. Moreover, it shows empirical evidence for the application of the Behavioral Perspective Model to digital consumption behavior in young users together with a methodology based on behavioral economics. The participants were 311 students in Colombia, where 50.48% were women. The factorial analysis of variance (ANOVA) allowed us to identify a statistically significant effect of the delay of the alternative reinforcer. However, there was no statistically significant effect of the interaction between the magnitude of the reinforcer and the delay time of the alternative reinforcer.

The manuscript “*The Vaccine Refusal. A Preliminary Interdisciplinary Investigation*”, by Brakel and Foxall, discusses vaccine refusal compared with two better-understood phenomena, namely, addiction and akrasia, along with the related matters of human action: intention, agency, will, and identity. Thus, vaccine refusal appears to be rewarded by “informational reinforcement”.

The manuscript “*Improving self-control: The influence of role models on the intertemporal choices of women*”, by Kedia et al., analyzes the extent to which delay-of-reward behaviors in female participants can be improved by observing others mastering it. The authors developed an intertemporal choice paradigm in which participants had to make fictitious choices between smaller rewards sooner and bigger ones later. In Study #1 (186 people), participants who delayed more had higher socioeconomic statuses and were less likely to procrastinate, smoke, or develop obesity. In Study #2 (178 people), female participants were exposed to a role model who, faced with intertemporal choices, mostly chose the delayed option. In Study #3 (148 people), the female participants chose the delayed option more often after having seen a high delay rather than a low delay model.

Finally, in the manuscript “*Introducing upfront money can decrease discounting in intertemporal choices with losses*”, by Jiang et al., the authors conducted two experiments and used hypothetical money rewards to examine whether the effect of upfront money could be extended to intertemporal choices with losses. The results showed that when both smaller-sooner (SS) and larger-later (LL) intertemporal losses were combined with an upfront loss or gain, people's discounting rate decreased and the preference for the SS option increased, thus supporting the salience account.

## Author contributions

All authors listed have made a substantial, direct, and intellectual contribution to the work and approved it for publication.

